# Kinetics of the non-neoplastic mucosa of the large bowel of dimethylhydrazine-treated rats.

**DOI:** 10.1038/bjc.1981.144

**Published:** 1981-07

**Authors:** J. P. Sunter, A. J. Watson, D. R. Appleton

## Abstract

Administration of 1,2 dimethylhydrazine (DMH) to rats by weekly s.c. injections causes the development of multiple epithelial tumours of the large bowel. These appear to arise as localized dysplastic abnormalities in hitherto apparently morphologically normal crypts. This study was undertaken in order to examine cell proliferation in such apparently normal crypts of DMH-treated animals. A number of proliferative abnormalities are evident, including changes in the size of the crypts, changes in the disposition of proliferating cells within them and reduced cell-cycle times. The nature and the extent of the abnormalities vary from site to site along the length of the bowel, and reflect the vulnerability of the different segments of the bowel, not only to the carcinogenic effects of DMH, but also to short-term toxicity.


*Br. J. Cancer* (1981) **44**, 35

### KINETICS OF THE NON-NEOPLASTIC MUCOSA OF THE LARGE BOWEL OF DIMETHYLHYDRAZINE-TREATED RATS

J. P. SUNTER, A. J. WATSON AND D. R. APPLETON*

*From the Departments of Pathology and *Medical Statistics, The University of Newcastle upon Tyne*

Received 5 January 1981 Accepted 26 March 1981

**Summary.—Administration of 1,2 dimethylhydrazine (DMH) to rats by weekly s.c. injections causes the development of multiple epithelial tumours of the large bowel. These appear to arise as localized dysplastic abnormalities in hitherto apparently morphologically normal crypts. This study was undertaken in order to examine cell proliferation in such apparently normal crypts of DMH-treated animals.**

**A number of proliferative abnormalities are evident, including changes in the size of the crypts, changes in the disposition of proliferating cells within them and reduced cell-cycle times. The nature and the extent of the abnormalities vary from site to site along the length of the bowel, and reflect the vulnerability of the different segments of the bowel, not only to the carcinogenic effects of DMH, but also to short-term toxicity.**

ADMINISTRATION of the carcinogen 1,2 dimethylhydrazine (DMH) to rats eventually results in the development of neoplasms in many organs (Druckrey, 1970). When it is given by repeated s.c. injections, a conspicuous organotropism is evident, with a high frequency of epithelial tumours of the intestinal tract (both small intestine and large bowel) and an acceptably low frequency of tumours elsewhere (Druckrey *et al.*, 1967; Druckrey, 1970; Martin *et al.*, 1973). Adjustment of the dosage schedule of the chemical can result in at least one colonic tumour in almost every treated animal by 20–30 weeks after the start of DMH treatment (Ward, 1974; Pozharisski, 1975; Maskens, 1976; Sunter *et al.*, 1978*a*). The microscopic structure of the tumours closely resembles that of human colonic neoplasms, and the model has been used quite extensively in the study of various aspects of colonic carcinogenesis, including the early stages of the morphogenesis of colonic cancer.

It is generally considered that frank neoplasia develops from areas of epithelial dysplasia or *in situ* neoplasia, such as have been described in many different situations in both experimental and human pathology. There is, however, some doubt as to the condition of the epithelial mucosa from which such dysplastic or pre-invasive lesions arise. For instance, Pozharisski (1975) stated that the development of malignant growths was not preceded by diffuse or focal hyperplasia of the mucosa, but he described abnormalities in the distribution of proliferating cells in otherwise normal crypts. On the other hand Wiebecke *et al.* (1973) described "more or less localized mucosal hyperplasias" in the colonic mucosa of DMH-treated rats. In the elongated crypts, the distribution of proliferating cells remained normal, but it was in such crypts that the earliest features of neoplasia were found. A progressive state of crypt hyperplasia, becoming more conspicuous with increasing duration of DMH treatment, has also been described (Maskens, 1976).

We have observed in the small-intestinal

Correspondence: Dr J. P. Sunter, Department of Pathology, Queen Elizabeth Hospital, Gateshead, Tyne and Wear, NE9 6SX.

mucosa of DMH-treated rats a state of crypt hyperplasia which occurs separately from and apparently before the development of frankly neoplastic changes, and we have previously characterized this lesion in cytokinetic terms (Sunter *et al.*, 1978*b*). In the present study we have used the same kinetic techniques to examine the non-neoplastic colonic mucosa of DMH-treated rats, in order to explore the possibility of proliferative changes in such mucosa. The results have been compared with those for a historical control group of normal rats from the same colony (Sunter *et al.*, 1979) and with those for DMH-induced colonic adenocarcinoma (Sunter *et al.*, 1980).

### MATERIALS AND METHODS

*Animals and DMH-treatment schedule.*—Randomly bred virgin female albino Wistar Porton rats from our own colony were used throughout. At the beginning of DMH treatment the animals were aged 12–16 weeks and weighed 250–300 g. They were fed on standard rat cake (N. E. Farmers, Aberdeen) and allowed tap water *ad libitum*.

A solution of symmetrical 1,2 dimethylhydrazine dihydrochloride (Aldrich Chemical Co.,) was administered by weekly s.c. injection, the dose being 15 mg (base)/kg body weight. The chemical was dissolved at a concentration of 1·66 g (of the dihydrochloride)/100 ml of normal saline, which contained 1·5% EDTA added as a stabilizer (Druckrey, 1970). The solution, freshly prepared each week, was brought to a pH of 6·4 by the addition of N NaOH.

At various times between 23 and 27 weeks after the start of DMH injections, animals were killed by cervical dislocation. In order to avoid distortions arising from the acute toxic effects of DMH on crypt cells, an interval of at last 1 week was allowed to elapse between the final injection of DMH and killing.

*Crypt morphometry, labelling and mitotic indices.*—Twenty-three weeks after the start of DMH injections 3 animals were killed, and a further 2 at 27 weeks. One hour before death, the animals had been given a single i.p. injection of tritiated thymidine [$^3$H]dT; Radiochemical Centre, Amersham, England) at a dose of 0·5 mCi/kg body weight; the specific activity of the [$^3$H]dT was 5 Ci/mmol. A full necropsy was performed on each animal, and the large bowel together with the caecum was removed, opened along its length and cleaned of faeces, then pinned mucosal surface uppermost to a cork board and placed in Carnoy's fixative for 6 h. The specimen was then transferred to 2 ethoxy-ethanol for a further 24 h before detailed inspection and dissection.

The total length of the colon was recorded, together with a description of the appearance and size of any tumours present, including a note as to their site. Transverse blocks of tumours were taken for histology and autoradiography, and the results have been reported elsewhere (Sunter *et al.*, 1978*a*; Sunter *et al.*, 1980). In addition to the blocks from the tumours, sections of tumour-free mucosa were obtained from 4 sites:

descending colon—30% of the distance from the anus to the ileocaecal valve;
transverse colon—60% of that distance;
ascending colon—90% of that distance;
caecum—the junction of the distal and middle thirds of the caecum.

These blocks were processed through to paraffin wax, and serial histological 3 $\mu$m sections were prepared, and routinely stained with haematoxylin and eosin. Autoradiographs were prepared using a dipping technique; exposure was for 4 weeks, and following development the slides were stained with Harris's haematoxylin.

For each sample the "left sides" of 100 axial crypt sections were analysed, provided that gross inspection of the intact bowel and microscopic examination of the sections showed no evidence of a neoplasm capable of producing local distortions of crypt architecture. The total length of the crypt column in cells was recorded, together with the positions of mitotic figures and labelled epithelial-cell nuclei. The criterion for a labelled nucleus was 5 or more autoradiographic grains located directly over it. Using a modification (Wright *et al.*, 1972) of the method of Cairnie & Bentley (1967) diagrams showing the mean labelling and mitotic indices at each cell position in a crypt of mean length were produced for each sample.

*Vincristine study.*—After 24 weeks of carcinogen treatment 7 animals were given vincristine sulphate (Oncovin, Eli Lilly) by i.p.

injection at 09:00 at a dosage of 1 mg/kg body weight. Individual animals were then killed at 20 min intervals up to 140 min after the injection. Blocks were taken as before from tumour-free mucosa and serial sections were prepared and stained with haematoxylin and eosin. The "left sides" of 100 axial crypt sections were analysed, care being taken to avoid the immediate neighbourhood of tumours. The positions of arrested metaphases were recorded, together with the total length of the crypt column in cells. The adequacy of metaphase arrest was confirmed by the absence of post-metaphase figures in the material studied. The data were projected on to a standard crypt, the height of which was the mean for that particular site in the bowel. Thus a series of mitotic index distribution diagrams was obtained showing the accumulation of arrested metaphases at the various times after vincristine administration.

From the analysis of 200 cross-sections of crypts containing metaphases (50 cross-sections in each of 4 animals) the mean crypt-column count (the number of cell nuclei forming the circumference of the crypt) was calculated for each of the 4 selected sites in the bowel. From the same material, the correction factor to compensate for overestimation of mitotic index due to analysis of axially sectioned crypts was calculated (Tannock, 1967).

*Fraction of labelled mitoses* (*FLM*) *study.*—A further group of 31 animals at their 24th week of DMH treatment were given an i.p. injection of [$^3$H]dT at 0·5 mCi/kg body weight at 09:00, and single animals were killed at hourly intervals up to 14 h after the injection, and thereafter at 2 hourly intervals up to 48 h. Histological material was dealt with as described previously. For analysis of the autoradiographs the crypt was divided into cell-position groups each consisting of 4 consecutive cell positions; the lowest group consisted of the first 4 cell positions at the base of the crypt, the second group Positions 5 to 8, and so on up the crypt. In each sample at least 20 mitotic figures were located in each cell position group and the proportion of mitoses showing $^3$H-labelling was determined. Thus an FLM curve for each cell position group was constructed. In the ascending colon it was impracticable to analyse crypts in this way because of the low mitotic index in the lowest cell positions, so the crypt was simply divided into an upper and lower half. At each site in the bowel an FLM curve for the whole crypt epithelium was constructed from the means of the values obtained in each of the component cell-position groups. The FLM curves were analysed by a modification of the computer method of Gilbert (1972).

### RESULTS AND INTERPRETATION

A considerable number of samples were found on microscopic examination to include a part of a carcinomatous tumour or an adenomatous polyp. Because of local perturbations of crypt architecture that these lesions might have induced, such samples were excluded from morphometric and kinetic analysis. In addition, many other samples included crypts which showed obvious changes of epithelial dysplasia or even *in situ* neoplasia, such as have been described by other workers. These obviously abnormal crypts were also excluded from the analysis. The following observations therefore can be taken to apply only to colonic mucosa between tumours where the crypts have an essentially normal architecture and evidence of abnormality of cellular differentiation is minimal.

The morphometric data relevant to the crypts as a whole are summarized in Table I, along with the corresponding values we have observed previously in

TABLE I.—*Crypt morphometry and mitotic index* (*normal values in parentheses*)

| Crypt parameters | Descending colon | Transverse colon | Ascending colon | Caecum |
|---|---|---|---|---|
| Mean lengths (cells) | 46·5 (41·8) | 50·4 (43·0) | 29·6 (33·2) | 31·1 (32·8) |
| Mean column count (cells) | 22·2 (17·6) | 22·0 (17·1) | 22·6 (19·0) | 24·6 (23·2) |
| Mean cell populations (cells) | 1030 (735) | 1110 (735) | 670 (630) | 765 (760) |
| Observed mitotic index | 0·23 (0·56) | 0·61 (0·48) | 0·28 (0·59) | 0·54 (0·55) |
| Tannock's factor | 0·62 (0·63) | 0·59 (0·63) | 0·57 (0·63) | 0·58 (0·56) |
| Corrected mitotic index | 0·14 (0·35) | 0·36 (0·30) | 0·16 (0·37) | 0·31 (0·31) |

TABLE II.—*Kinetic data for the whole crypt calculated for the vincristine study and the mitotic index* (*normal values in parentheses*)

| Parameter | Descending colon | Transverse colon | Ascending colon | Caecum |
|---|---|---|---|---|
| Birth rate (cells/1000/h) | 14 (10) | 10 (13) | 10 (12) | 17 (15) |
| Mitosis per crypt column | 0·067 (0·15) | 0·181 (0·13) | 0·048 (0·12) | 0·098 (0·10) |
| Cumulative $k_B$ (cells/col/h) | 0·67 (0·42) | 0·51 (0·51) | 0·31 (0·34) | 0·50 (0·43) |
| Cell production rate (cells/h) | 14·9 (7·3) | 11·2 (8·7) | 7·0 (6·5) | 12·3 (9·9) |
| Mitotic duration (h) | 0·10 (0·36) | 0·35 (0·25) | 0·15 (0·35) | 0·20 (0·23) |

normal animals from our colony (Sunter *et al.*, 1979). In the descending colon and transverse colon, treated animals show increases in both length and circumference of crypts, leading to a considerably increased cell population. In the ascending colon there are only minor changes in crypt length and circumference which do not change the overall size of the crypt cell population; in the caecum no significant changes are apparent. The means for the observed mitotic indices for the crypt as a whole show interesting deviations from the values obtained in normal animals. In the descending and ascending colon the indices are half normal, while those in the transverse colon and caecum show no obvious change. There are only minor changes in Tannock's factor, and the mitotic indices, corrected to take into account the geometric artefact introduced by the counting of axially sectioned crypts, confirm these results.

Table II shows the results from a consideration of the data derived from the stathmokinetic experiment and the corrected mitotic indices. For each site in the bowel the corrected whole-crypt mitotic index was plotted against time after vincristine. A rectangular age distribution was assumed for the crypt as a whole and a straight line was fitted by least squares. From the slopes of these lines, estimates of the cell birth rate were derived; they are similar to those we have obtained previously in normal rats, and this is perhaps surprising in view of the considerable reduction in mitotic index in treated animals in descending and ascending colon. The estimates of cumulative mitotic index in cells/column can be obtained from the product of mitotic index and mean crypt length; the fluctuations reflect changes in these variables.

From the distribution of arrested metaphases along the length of the crypt, at various times after the administration of vincristine, the accumulation of metaphases at each individual cell position has been monitored. We have assumed an exponential age distribution at individual cell positions; the estimates of cell birth rate have then been summed to yield a cumulative rate at the top of the crypt column (de Rodriguez *et al.*, 1979). This is the number of cells produced per column per hour, and is similar in treated and in normal animals, save that in descending colon it is somewhat increased in treated animals. The rate of cell production for the crypt as a whole can be evaluated as the product of cumulative cell birth rate and column count, and is shown in Table II; also shown are estimates of mitotic duration (de Rodriguez *et al.*, 1979). These show reductions in the duration of mitosis in descending and ascending colon, which would explain some of the apparent inconsistencies in the data.

The cell-cycle phase durations calculated from the curves fitted to the whole-crypt FLM data are shown in Table III. The durations of the S ($t_S$) and of the $G_2$ phase ($t_{G2}$) are not significantly altered from normal, but reduction in total cell-cycle time ($T_c$) has occurred at all 3 sites in the colon, owing to a reduction in the length of $G_1$ ($t_{G1}$). Despite these changes, the $T_c$ values at the 3 colonic sites retain their normal gradient, with the shortest $T_c$ in the ascending colon, an intermediate value in the transverse colon, and the longest in the descending colon. We have previously used the FLM technique to study

TABLE III.—*Kinetic data for the whole crypt derived principally from [$^3H$]dT labelling studies (normal values in parentheses)*

| | Descending colon | Transverse colon | Ascending colon | Caecum |
|---|---|---|---|---|
| $t_{G1}$ (h) | 27·8 (46·6) | 21·3 (30·7) | 15·4 (24·6) | 16·6 (15·2) |
| $t_S$ (h) | 8·9 (9·0) | 8·3 (9·1) | 10·3 (8·8) | 7·9 (8·5) |
| $t_{G2}$ (h) | 2·4 (2·0) | 2·2 (2·0) | 1·4 (1·6) | 1·8 (1·5) |
| $T_c$ (h) | 39·1 (57·9) | 31·9 (42·0) | 27·1 (39·3) | 26·4 (25·4) |
| Coefficient of variation of $T_c$ | 45 (52) | 37 (38) | 29 (54) | 31 (26) |
| Corrected theoretical labelling index | 28 (21) | 30 (24) | 41 (32) | 33 (26) |
| Observed labelling index | 5·5 (7·1) | 8·8 (7·2) | 5·0 (7·3) | 9·5 (10·9) |
| Growth fraction (%) | 20 (34) | 30 (30) | 12 (23) | 28 (30) |
| Total no. of proliferating cells/crypt | 205 (250 | 325 (220) | 80 (145) | 220 (230) |
| Half-maximum position | 20 (14) | 29 (26) | 19 (20) | 13 (13) |
| Cut-off position | 10 (11) | 21 (17) | 14 (16) | 9 (10) |

cell proliferation in the various histological types of colonic tumour in DMH-treated rats; the mean $T_c$ values were all around 20 h (Sunter *et al.*, 1980)—even less than the estimates obtained in the present study on apparently healthy mucosa in DMH-treated rats.

Also shown in Table III are the estimates of the coefficient of variation of $T_c$ provided by the Gilbert (1972) programme, and estimates of the theoretical labelling indices. In the calculation of this latter parameter we have assumed a rectangular age distribution, and have compensated for the variability of $T_c$ (Appleton & Sunter, 1979). Mean labelling indices for the pulse-labelled animals are also shown, and the estimates of growth fraction (the proportion of cells proliferating) derived from the ratio of observed to theoretical labelling indices. These results show reductions in observed labelling index and growth fraction in descending and ascending colon. From the product of growth fraction and crypt-cell population the number of proliferating cells per crypt can be calculated. In descending and ascending colon there is a reduction in the number of proliferating cells per crypt (which in the descending colon has occurred despite a moderate increase in the actual size of the crypt), while the enlarged crypts in the transverse colon show a proportionate increase in the number of proliferating cells.

An indication of the distribution of proliferating cells along the length of the crypt is obtained from the labelling-index distribution diagrams which have been constructed for each sample taken from the group of pulse-labelled animals. The figure shows the pooled data for all 5 animals for each of the 4 sites in the bowel. The shaded areas indicate 95% confidence limits for the points obtained by pooling data from a group of 4 control animals. All the individual samples from a particular site showed broadly similar features. In the descending colon, where the labelling index as a whole is somewhat reduced in treated animals, it appears that the distribution of labelled cells is altered, with a reduction in labelling index throughout the proliferation zone and extension of labelled cells into higher cell positions within the slightly lengthened crypt. The pattern of a lower labelling index in the basal cell positions is lost. In the transverse colon where labelling index as a whole is somewhat enhanced, it is in the highest (and to some extent the lowest) cell positions that the increased numbers of labelled cells are found. In the ascending colon there is a fairly symmetrical small reduction in labelling index, and in the caecum no change is seen, as might be anticipated in view of the absence of any other significant changes. An indication of the size of the compartment within the crypt where there are proliferating cells

FIGURE.—Labelling-index distribution for (i) descending colon, (ii) transverse colon, (iii) ascending colon and (iv) caecum, obtained by pooling the data from 5 pulse-labelled animals. The shaded areas indicate 95% confidence limits for the points obtained by pooling data from a group of 4 control animals.

may be gained by the position at which labelling index is reduced to half its maximum value (Cleaver, 1967) or by the cut-off position (Cairnie *et al.*, 1965; Appleton *et al.*, 1981) and both these parameters are estimated in Table III.

The estimates of the cell-cycle times ($T_c$) calculated for the separate four-cell position groups are shown in Table IV, along with the corresponding estimates we have obtained previously in normal animals. $T_c$ is consistently reduced in every cell position group at all 3 sites in the colons of the treated animals. This consistency is reassuring in view of the unreliability of the standard errors estimated by the Gilbert programme. In the caecum there is no convincing evidence for a change. In those sites where changes are evident the usual distribution of $T_c$ within the crypt is still retained, the longest times occurring at the base of the crypt.

We have been dealing with differences in parameter estimates between treated and control rats in terms of their size, and not of their statistical significance. It is indeed difficult to assess the standard errors of some of the quantities estimated, such as the cut-off position, or the cell-cycle-phase durations, and thus the theoretical labelling index and growth fraction; but for the more straightforward variables we can perform *t* tests between the means of treated and control groups. For example the increase in crypt length in descending colon is 4·7 cells ± 0·97 (s.e., calculated from 14 control animals and 11 treated)—a significant increase.

TABLE IV.—*Cell-cycle times (h) at different sites along the length of the bowel, calculated by cell-position groups within the crypt (normal values in parentheses)*

| Cell-position group | Descending colon | Transverse colon | Ascending colon | Caecum |
|---|---|---|---|---|
| 1–4 (base) | 41·4 (68·4) | 53·0 (70·7) | | 30·2 (34·6) |
| 5–8 | 40·0 (61·0) | 37·5 (64·4) | 28·2 (39·7) | 25·1 (25·4) |
| 9–12 | 35·5 (64·9) | 45·2 (39·8) | | 23·8 (23·4) |
| 13–16 | 38·4 (45·9) | 32·8 (38·5) | | 23·4 (24·1) |
| 17–20 | 36·6 (53·5) | 29·9 (38·7) | 26·9 (32·1) | |
| 21–24 | 33·5 (71·5) | 27·0 (37·4) | | |
| 25+ | | 26·4 (—) | | |

The estimate of the cumulative cell-birth rate has a standard error of 0·03 in descending colon of both control and treated rats, if we ignore any possible error in the estimation of Tannock's factor and are satisfied as to the adequacy of metaphase accumulation and of the assumption of an exponential age distribution at each cell position. Trying to take the non-systematic elements of these other sources of error into account, *e.g.* by postulating a relative standard error of 10% in Tannock's factor, increases the standard error of the difference, but this in fact still leaves the difference (0·67–0·42 = 0·25) significantly different from zero, the new estimate of its standard error being 0·8. This indicates that the size of the experiment is such that differences which look worthy of discussion probably also attain statistical significance.

### DISCUSSION

In the normal rat the morphology and cytokinetic organization of the mucosal crypts vary quite considerably from one site to another in the large bowel and the caecum (Sunter *et al.*, 1979). Additionally, the various segments of the bowel show different degrees of vulnerability to the long-term carcinogenic effects of chemicals of the cycasin group (Pozharisski, 1975; Maskens, 1976; Nomura *et al.*, 1978; Sunter *et al.*, 1978*a*). These two considerations make the assessment of the possible preneoplastic nature of the changes in the mucosal crypts rather complex. Further problems are caused by the appearance of lesions variously described as dysplasias, adenomatous changes in glands, and *in situ* neoplasias, which are generally considered to be the immediate precursors of frank neoplasms. We have tried to exclude such lesions in this study, which has been designed to document any generalized changes in apparently normal areas of mucosa in DMH-treated rats.

### *Changes in size crypt*

Pozharisski (1975) stated that in his experience the abnormal mucosal crypts in the large bowel of DMH-treated rats were of a normal length, and could not therefore be properly regarded as hyperplastic. Wiebecke *et al.* (1973) and Ward (1974) described focal hyperplastic lesions in the colon, from which early neoplasms appeared to originate. On the other hand Maskens (1976), using material from the descending colon, showed a steady and progressive increase in the length of the mucosal crypts, which appeared diffuse. Again, using material from the descending colon, Tutton & Barkla (1976) found that, while crypt length did not alter significantly during DMH treatment, there was a considerable increase in crypt circumference, leading to an increase in cell population from 1300 to 1800 cells. In the present study we have found increases in crypt length and crypt-column count in transverse colon and descending colon, with an increase in population size similar to that observed by Tutton & Barkla (1976). The minor changes we have noted in the ascending colon effect no significant change in crypt-population size, and in caecum there is little evidence of change in any of these parameters. In the consideration of overall crypt size, it is of note that Nomura *et al.* (1978) described areas of mucosal atrophy in addition to areas of hyperplasia.

*Distribution of proliferating cells within the crypt*

While a change in crypt length was not a feature in the material of Pozharisski (1975), his studies showed a considerable lengthening of the zone in which $^3$H-labelled cells were seen. This increase in the absolute size of the proliferation zone has been commented on by others (Wiebecke *et al.*, 1973; Maskens, 1976; Tutton & Barkla, 1976). Increased labelling indices were apparent in the study of Wiebecke *et al.* (1973) but not in that of Maskens (1976). In our material too there appears to be an increase in the absolute size of the proliferation zone, as evidenced by the changes in half-maximum position in the descending and transverse colon. Whole-crypt labelling indices are reduced in the ascending and descending colon but perhaps increased in the transverse colon. The net results of the changes are essentially a loss of the normal pattern of a lower index in basal cell positions than higher up the crypt in transverse and descending colon, and a slight symmetrical reduction in the ascending colon. The caecum does not show any significant change.

*Rates of cell proliferation*

In the descending and ascending colon the mitotic index is much lower in treated animals than in controls. This, coupled with the data derived from the stathmokinetic experiment with vincristine, used to calculate a cumulative birth rate, suggests a surprising fall in the duration of mitosis in these 2 sites. This rather unexpected finding will require further investigation in subsequent studies.

Crypt cell production rate, a measure of the net output of cells per crypt, is clearly increased in the descending colon, but the significance of the changes in transverse colon and caecum is much less certain.

The FLM experiment shows a clear and consistent pattern of a reduced cell-cycle time ($T_C$) in treated mucosa at all 3 colonic sites. The reduction is effected through $G_1$, other cell-cycle phases showing no great changes. Analysis of the data by cell-position groups shows that cells at all levels in the crypt are affected similarly, but that the usual distribution $T_C$ within the crypt is retained. Overall, the values for $T_C$ in the DMH-treated mucosa are intermediate between those of the normal mucosa and those of DMH-induced colonic tumours (Sunter *et al.*, 1980). The estimates of growth fraction in the whole crypt show reductions in ascending colon, with no change in the transverse colon. Given that the overall size of the proliferation compartment is increased in descending and transverse colon and unchanged in the ascending colon, the figures suggest that the reduction in the growth fraction of whole crypt is largely a result of a fall within the proliferation zone itself, a situation we have previously observed in the small bowel mucosa of DMH-treated rats (Sunter *et al.*, 1978*b*).

### CONCLUDING REMARKS

This study has shown that there are a number of morphometric and cytokinetic abnormalities in the morphologically normal-looking crypts in the large-bowel mucosa of rats exposed to chronic DMH. The most obvious abnormality is an increase in the numbers of cells in the crypts, which is confined to the transverse colon and the descending colon; in the ascending colon, although there are morphometric abnormalities, no significant alteration in crypt-cell population is seen, and in the caecum there is no change in morphometric indices. Changes in the distribution of proliferating cells are apparent in transverse and descending colon. The normal patern of a low labelling index in the basal cell-position groups becomes less obvious and the proliferation zone extends further up the crypt, both in absolute and relative terms. Again the ascending colon is less severely affected, only a slight general reduction being apparent. No significant abnormality is seen in the caecum. The severity of these changes seems to reflect very closely the vulnerability of the intestinal mucosa at different sites to the chronic carcinogenic effects of DMH; in our material caecal tumours are virtually never seen, tumours in the proximal part of the ascending colon are rare and tumours in the rest of the bowel are very common (Sunter *et al.*, 1978*a*). Similar selective vulnerability to the acute toxic effects of DMH is seen (Sunter *et al.*, 1981).

At all 3 colonic sites there is a substantial and consistent reduction in cell-cycle time, brought about by a reduction in the duration of $G_1$. The growth fraction is reduced in descending and ascending colon, while in the transverse colon the estimate is the same as for normal animals. However, given the changes in the size of the proliferation zone, it is apparent that even at this site there must be a substantial reduction in growth fraction within the proliferation zone, just as in descending and ascending colon. The caecum shows no obvious changes of proliferative indices calculated from thymidine-labelling studies.

It is a matter for speculation whether the kinetic abnormalities described represent a specific preneoplastic state or a nonspecific response to a toxin; work is in progress to try to define these mucosal changes using other techniques.

This work was supported by a grant from the North of England Council of the Cancer Research Campaign. We would like to thank Miss E. Wark and Miss Y. Allen who typed successive versions of the manuscript, Mrs E Wallace who provided technical assistance, and Mr R. Joyce who prepared the illustration.

### REFERENCES

APPLETON, D. R. & SUNTER, J. P. (1979) Estimating the proportion of proliferating cells in a population. *Virchows Arch.* [*Cell Pathol.*], **32**, 69.

APPLETON, D. R., SUNTER, J. P. & WATSON, A. J. (1981) The cut-off position in the intestinal crypt. *Cell Tissue Kinet.*, (in press).

CAIRNIE, A. B. & BENTLEY, J. (1967) Cell proliferation studies in the intestinal epithelium of the rat: Hyperplasia during lactation. *Exp. Cell Res.*, **46**, 428.

CAIRNIE, A. B., LAMERTON, L. F. & STEEL, G. G. (1965) Cell proliferation studies in the intestinal epithelium of the rat. II. Theoretical aspects. *Exp. Cell Res.*, **39**, 539.

CLEAVER, J. E. (1967) *Thymidine Metabolism and Cell Kinetics*. Amsterdam: North Holland.

DE RODRIGUEZ, M. S. B., SUNTER, J. P., WATSON, A. J., WRIGHT, N. A. & APPLETON, D. R. (1979) Cell population kinetics in the mucosal crypts of the descending colon of the mouse. *Virchows Archiv.* [*Cell Pathol.*], **29**, 351.

DRUCKREY, H. (1970) Production of colonic carcinomas by 1,2 dialkyhydrazines and azoxyalkanes. In *Carcinoma of the Colon and Antecedent Epithelium*. Ed. Burdette. Springfield: Thomas. p. 267.

DRUCKREY, H., PREUSSMAN, R., MATZKIES, F. & IVANKOVIC, S. (1967) Selektive Erzeugung von Darmkrebs bei Ratten durch 1,2 Dimethylhydrazin. *Naturwissenschaften*, **54**, 285.

GILBERT, C. W. (1972) The labelled mitoses curve and the estimation of the parameters of the cell cycle. *Cell Tissue Kinet.*, **5**, 53.

MARTIN, M. S., MARTIN, F., MICHIELS, R., BASTEIN, H., JUSTRABO, E., BORDES, M. & VIRY, B. (1973) An experimental model for cancer of the colon and rectum. *Digestion*, **8**, 22.

MASKENS, A. P. (1976) Histogenesis and growth pattern of 1,2 dimethylhydrazine induced rat colon adenocarcinoma. *Cancer Res.*, **36**, 1585.

NOMURA, K., SCHLAKE, W. & GRUNDMANN, E. (1978) New aspects of intestinal carcinogenesis by 1,2 dimethylhydrazine dihydrochloride (DMH) and the influence of antilymphocytic globulin (ALG) on its progress. *Z. Krebsorsch.*, **92**, 17.

POZHARISSKI, K. M. (1975) Morphology and morphogenesis of experimental epithelial tumours of the intestine. *J. Natl Cancer Inst.*, **54**, 1115.

SUNTER, J. P., APPLETON, D. R. & WATSON, A. J. (1981) Acute changes occurring in the intestinal mucosae of rats given a single injection of 1,2 dimethylhydrazine. *Virchows Archiv.* [*Cell Pathol.*], **36**, 47.

SUNTER, J. P., APPLETON, D. R., WRIGHT, N. A. & WATSON, A. J. (1978*a*) Pathological features of the colonic tumours induced in rats by the administration of 1,2 dimethylhydrazine. *Virchows Archiv.* [*Cell Pathol.*], **29**, 211.

SUNTER, J. P., APPLETON, D. R., WRIGHT, N. A. & WATSON, A. J. (1978*b*) Kinetics of changes in the crypts of the jejunal mucosa of dimethylhydrazine-treated rats. *Br. J. Cancer*, **37**, 662.

SUNTER, J. P., HULL, D. L., APPLETON, D. R. & WATSON, A. J. (1980) Cell proliferation of colonic neoplasms in dimethylhydrazine-treated rats. *Br. J. Cancer*, **42**, 95.

SUNTER, J. P., WATSON, A. J., WRIGHT, N. A. & APPLETON, D. R. (1979) Cell proliferation at different sites along the length of the rat colon. *Virchows Archiv.* [*Cell Pathol.*], **32**, 75.

TANNOCK, I. F. (1967) A comparison of the relative efficiencies of various metaphase arrest agents. *Exp. Cell Res.*, **47**, 345.

TUTTON, P. J. M. & BARKLA, D. H. (1976) Cell proliferation in the descending colon of dimethylhydrazine treated rats and in dimethylhydrazine induced adenocarcinomas. *Virchows Archiv.* [*Cell Pathol.*], **21**, 147.

WARD, J. M. (1974) Morphogenesis of chemically induced neoplasms of the colon and small intestine in rats. *Lab. Invest.*, **30**, 505.

WIEBECKE, B., KREY, U., LÖHRS, U. & EDER, M. (1973) Morphological and autoradiographical investigations on experimental carcinogenesis and polyp development in the intestinal tract of rats and mice. *Virchows Archiv.* [*Pathol. Anat.*], **360**, 179.

WRIGHT, N. A., MORLEY, A. R. & APPLETON, D. R. (1972) Variation in the duration of mitosis in the crypts of Lieberkühn of the rat: A cytokinetic study using vincristine. *Cell Tissue Kinet.*, **5**, 351.


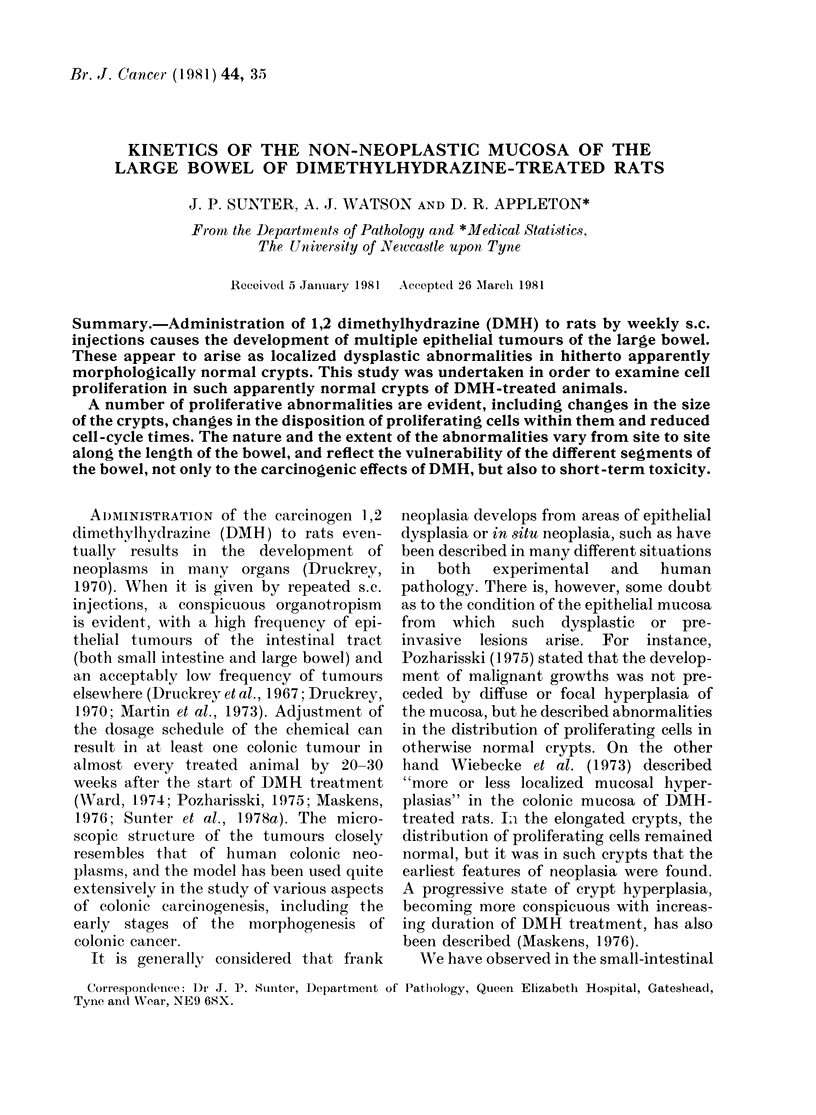

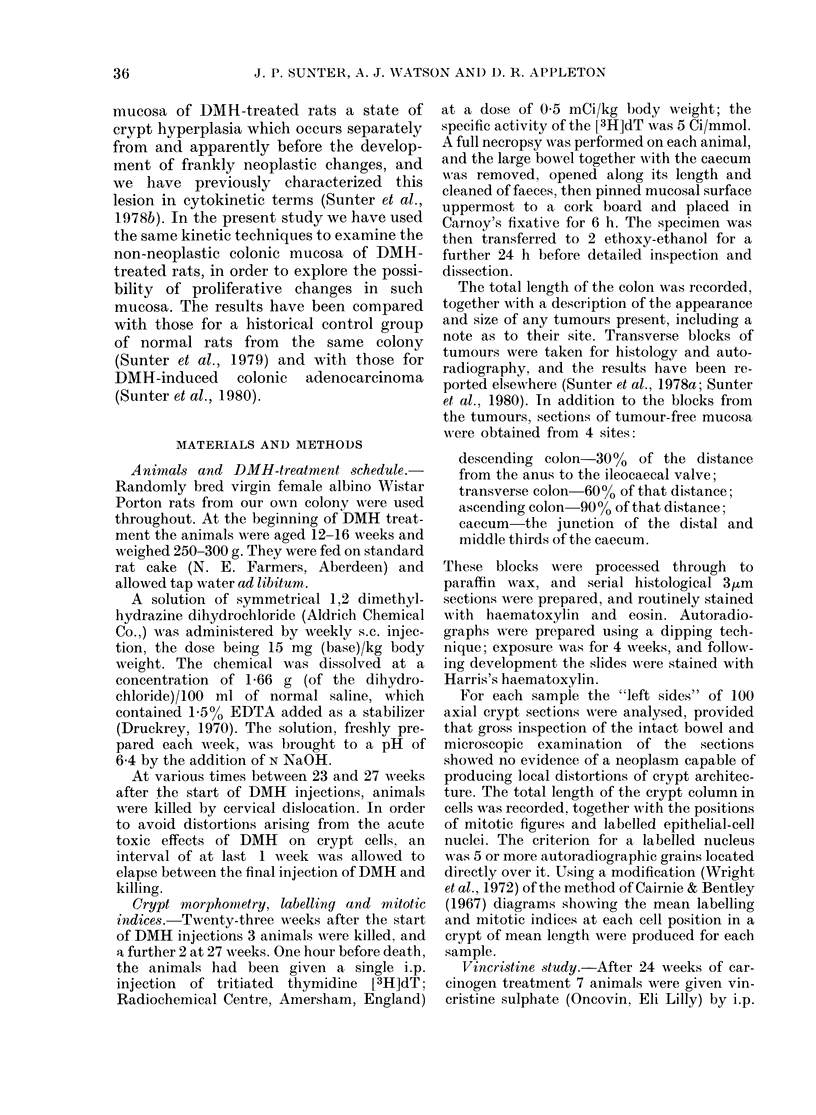

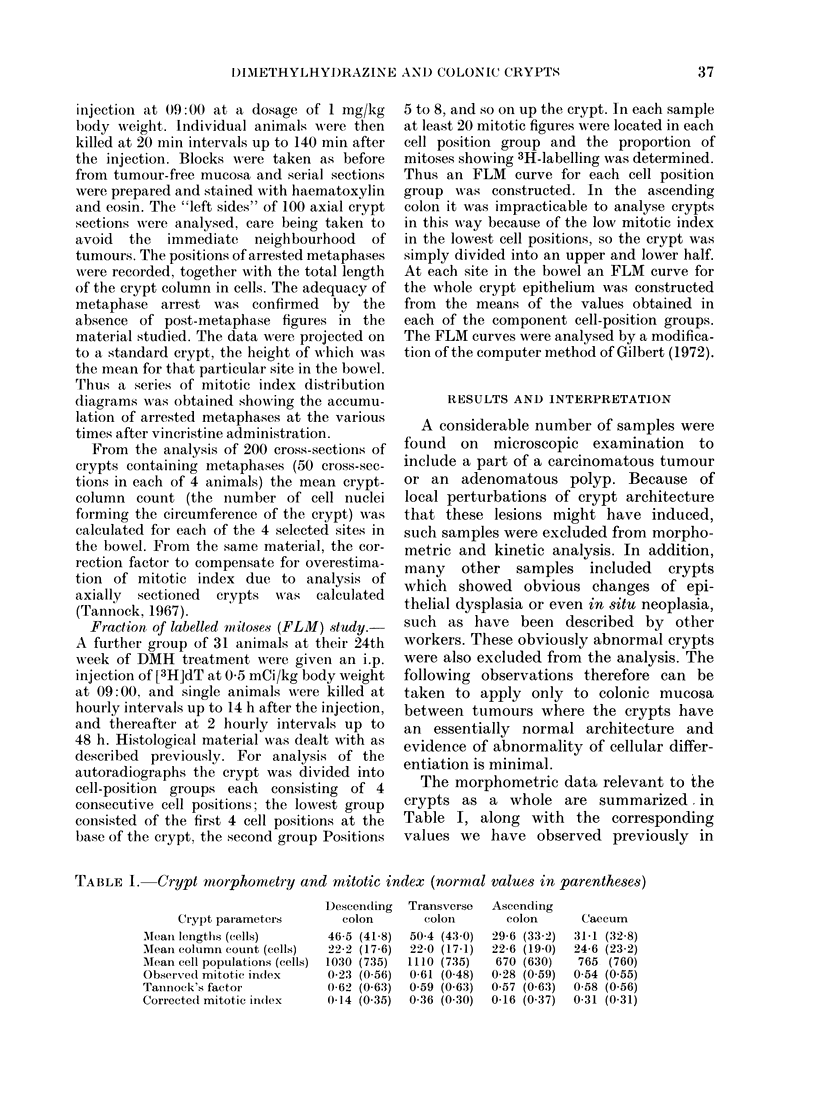

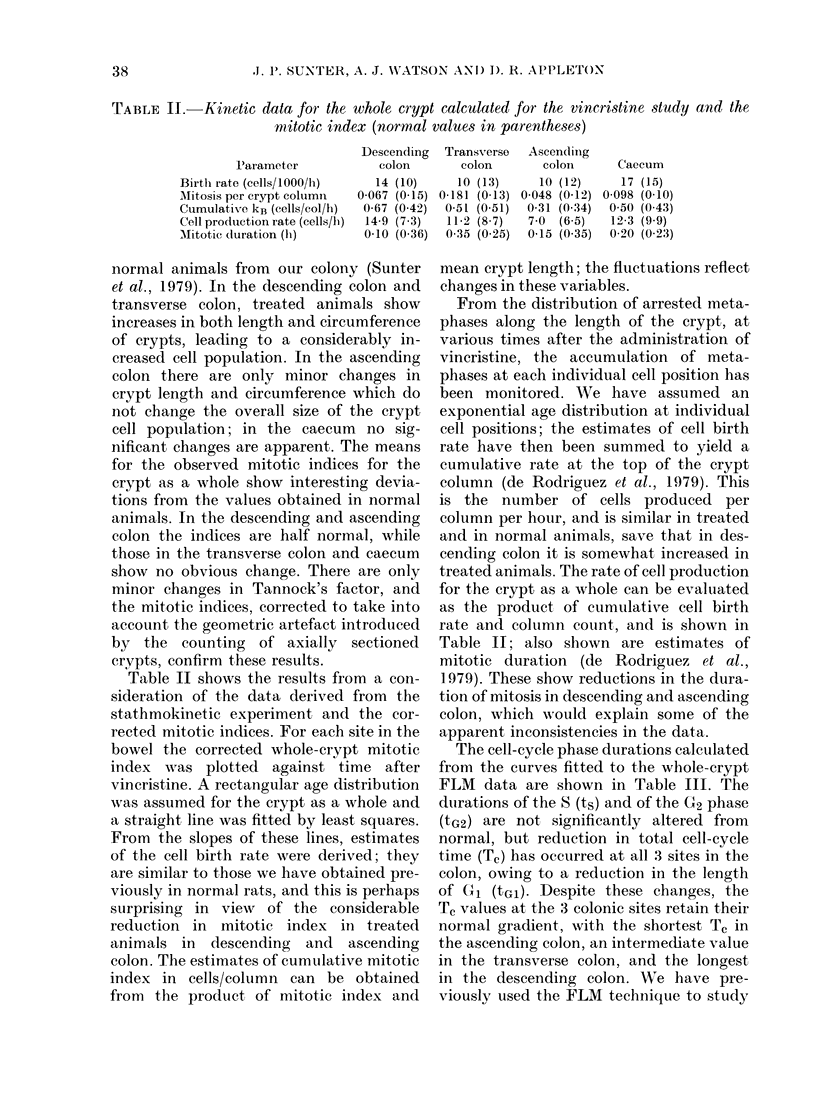

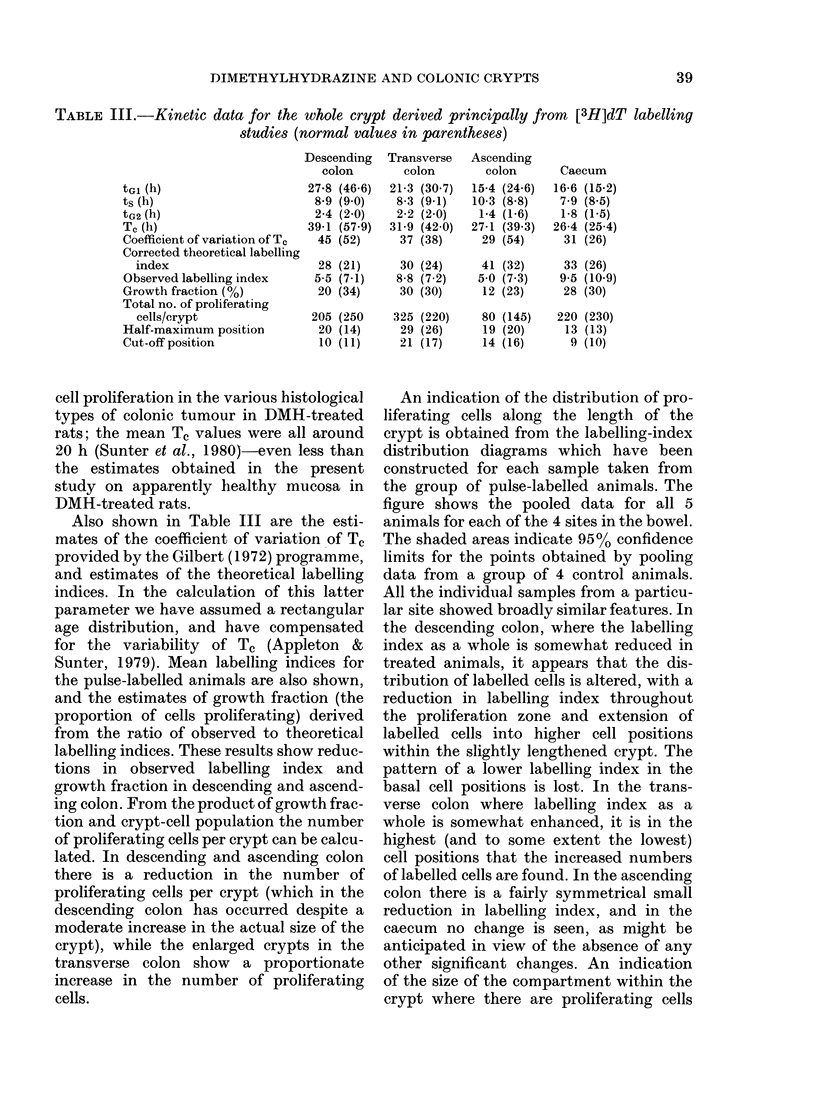

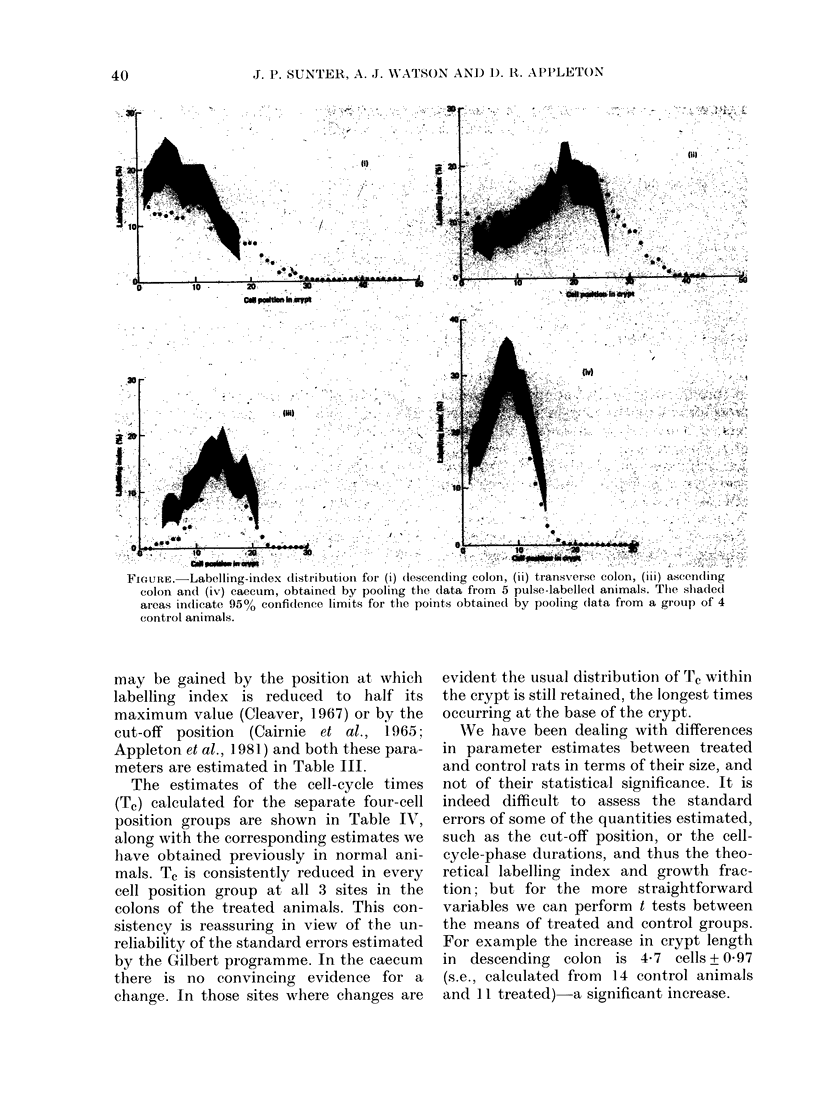

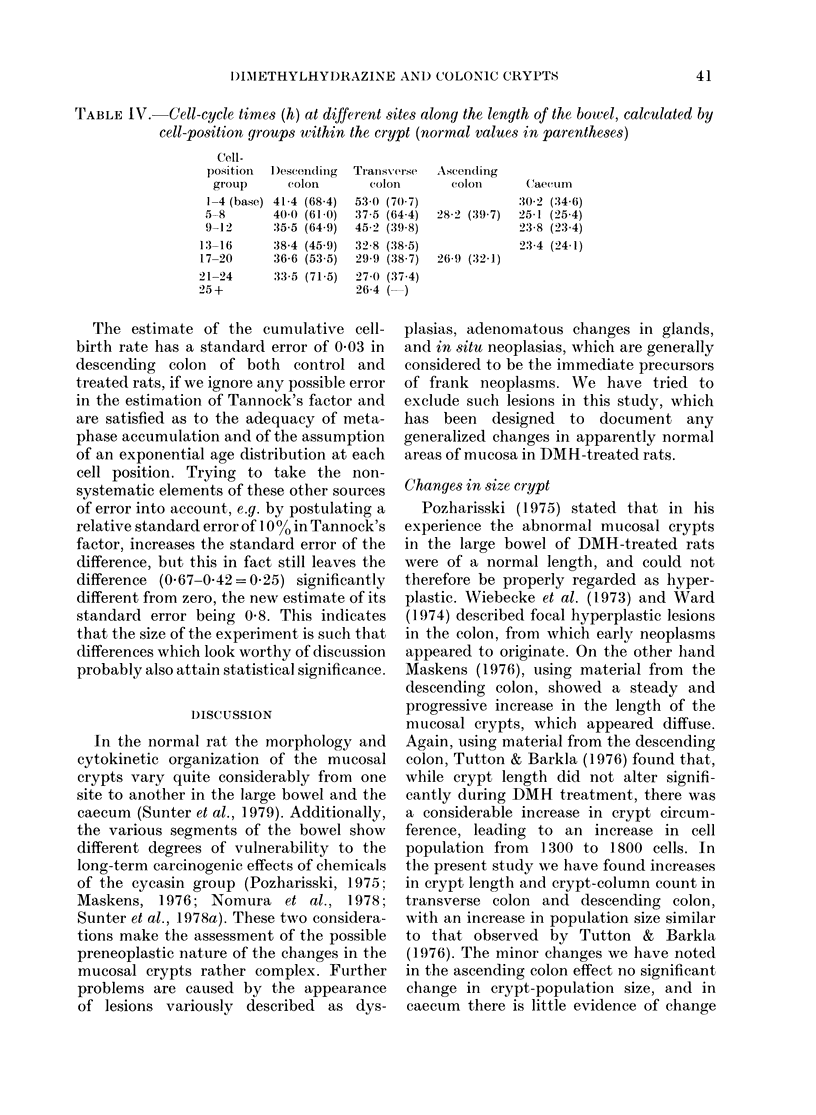

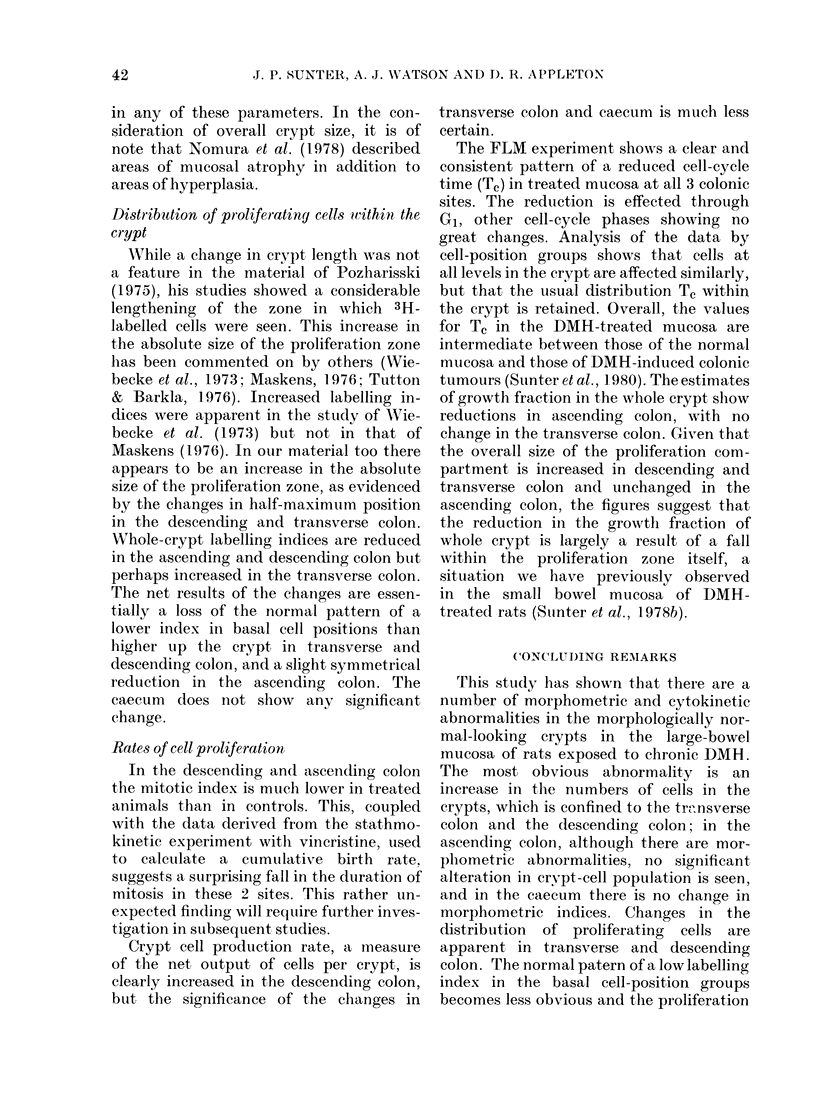

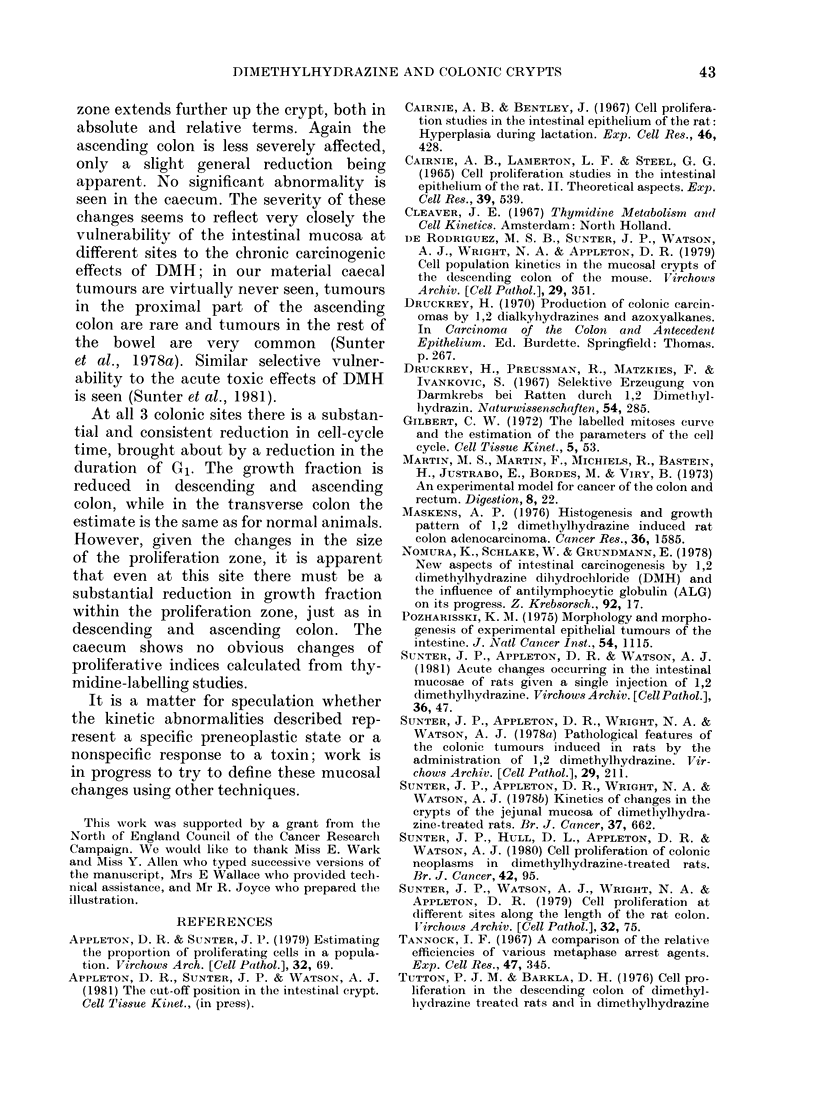

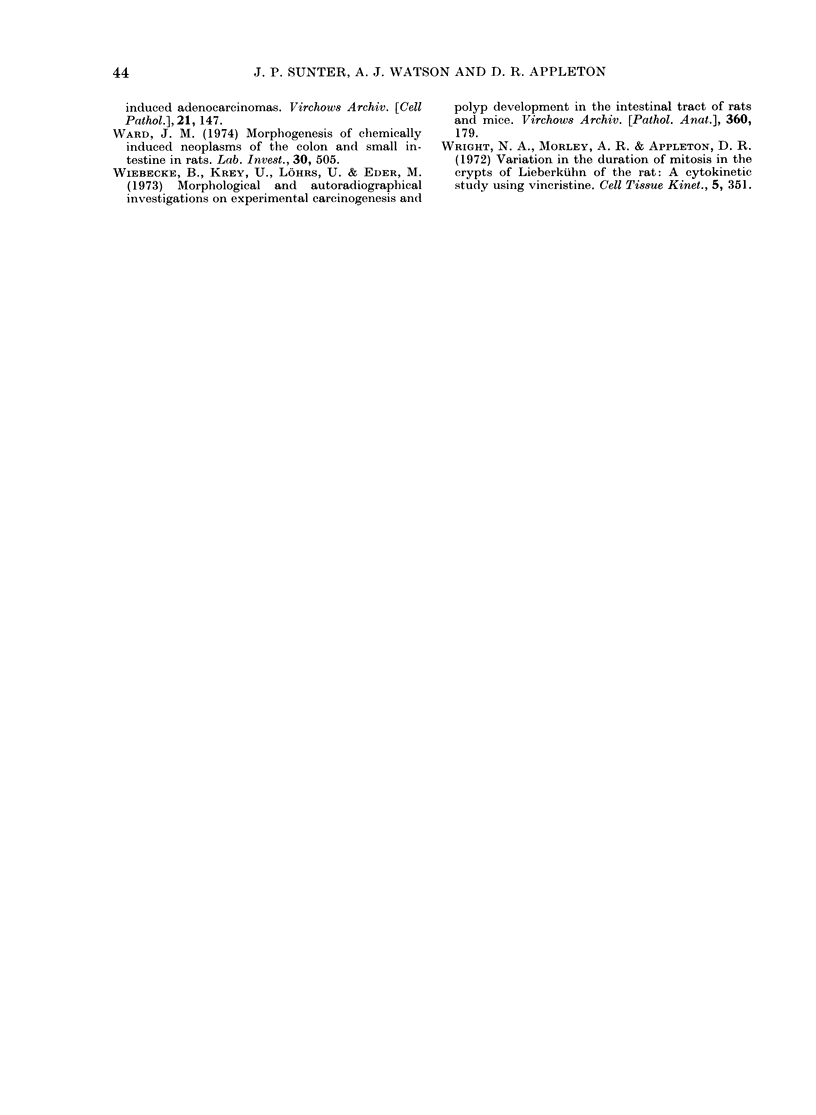


## References

[OCR_00963] Cairnie A. B., Bentley R. E. (1967). Cell proliferation studies in the intestinal epithelium of the rat. Hyperplasia during lactation.. Exp Cell Res.

[OCR_00969] Cairnie A. B., Lamerton L. F., Steel G. G. (1965). Cell proliferation studies in the intestinal epithelium of the rat. II. Theoretical aspects.. Exp Cell Res.

[OCR_00995] Druckrey H., Preussmann R., Matzkies F., Ivankovic S. (1967). Selektive Erzeugung von Darmkrebs bei Ratten durch 1,2-Dimethyl-hydrazin.. Naturwissenschaften.

[OCR_00999] Gilbert C. W. (1972). The labelled mitoses curve and the estimation of the parameters of the cell cycle.. Cell Tissue Kinet.

[OCR_01004] Martin M. S., Martin F., Michiels R., Bastien H., Justrabo E., Bordes M., Viry B. (1973). An experimental model for cancer of the colon and rectum. Intestinal carcinoma induced in the rat 1,2-dimethylhydrazine.. Digestion.

[OCR_01010] Maskens A. P. (1976). Histogenesis and growth pattern of 1,2-dimethylhydrazine-induced rat colon adenocarcinoma.. Cancer Res.

[OCR_01015] Nomura K., Schlake W., Grundmann E. (1978). New aspects of intestinal carcinogenesis by 1,2-dimethylhydrazine dihydrochloride (DMH) and the influence of antilymphocyte globuline (ALG) on its progress.. Z Krebsforsch Klin Onkol Cancer Res Clin Oncol.

[OCR_01024] Pozharisski K. M. (1975). Morphology and morphogenesis of experimental epithelial tumors of the intestine.. J Natl Cancer Inst.

[OCR_01041] Sunter J. P., Appleton D. R., Wright N. A., Watson A. J. (1978). Kinetics of changes in the crypts of the jejunal mucosa of dimethylhydrazine-treated rats.. Br J Cancer.

[OCR_01034] Sunter J. P., Appleton D. R., Wright N. A., Watson A. J. (1978). Pathological features of the colonic tumours induced in rats by the administration of 1,2-dimethylhydrazine.. Virchows Arch B Cell Pathol.

[OCR_01047] Sunter J. P., Hull D. L., Appleton D. R., Watson A. J. (1980). Cell proliferation of colonic neoplasms in dimethylhydrazine-treated rats.. Br J Cancer.

[OCR_01074] Ward J. M. (1974). Morphogenesis of chemically induced neoplasms of the colon and small intestine in rats.. Lab Invest.

[OCR_01079] Wiebecke B., Krey U., Löhrs U., Eder M. (1973). Morphological and autoradiographical investigations on experimental carcinogenesis and polyp development in the intestinal tract of rats and mice.. Virchows Arch A Pathol Pathol Anat.

[OCR_01088] Wright N., Morley A., Appleton D. (1972). Variation in the duration of mitosis in the crypts of Lieberkuhn of the rat; a cytokinetic study using vincristine.. Cell Tissue Kinet.

[OCR_00979] de Rodriguez M. S., Sunter J. P., Watson A. J., Wright N. A., Appleton D. R. (1979). Cell population kinetics in the mucosal crypts of the descending colon of the mouse.. Virchows Arch B Cell Pathol.

